# Calcium sensing receptor expression is downregulated in gastroenteropancreatic neuroendocrine tumours via epigenetic mechanisms

**DOI:** 10.1002/ijc.35264

**Published:** 2024-11-23

**Authors:** Katherine A. English, Michelle Goldsworthy, Brittannie Willis, Kreepa G. Kooblall, Shweta Birla, Andreas Selberherr, Mark Stevenson, Omair A. Shariq, Ann L. Oberg, Tony Wang, James Carmichael, Konstantinos Mavrommatis, Laure Escoubet, Rajesh V. Thakker, Sarah A. Howles, Kate E. Lines

**Affiliations:** ^1^ OCDEM, Radcliffe Department of Medicine University of Oxford Oxford UK; ^2^ Nuffield Department of Surgical Sciences University of Oxford Oxford UK; ^3^ Department of Surgery Medical University of Vienna Vienna Austria; ^4^ Department of Quantitative Health Sciences Mayo Clinic Rochester Minnesota USA; ^5^ Bristol‐Myers Squibb San Diego California USA; ^6^ Oxford NIHR Biomedical Research Centre Oxford University Hospitals Trust Oxford UK; ^7^ Centre for Endocrinology, William Harvey Research Institute, Barts and the London School of Medicine Queen Mary University of London London UK; ^8^ School of Biological and Medical Sciences, Faculty of Health and Life Sciences Oxford Brookes University Oxford UK

**Keywords:** calcium signalling, epigenetics, gastrinoma, insulinoma, non‐functioning PNET

## Abstract

Gastroenteropancreatic neuroendocrine tumours (GEP‐NETs), which may be hormone secreting (e.g., gastrinomas and insulinomas) or non‐secreting (also known as non‐functioning NETs) are associated with severe morbidity and have a median overall survival of 75–124 months. Studies have highlighted the importance of epigenetic mechanisms in GEP‐NETs pathogenesis, with the most frequently mutated genes being the epigenetic regulators, *MEN1*, *DAXX*, and *ATRX*. However, the consequences of these aberrant epigenetic mechanisms are poorly understood. The calcium sensing receptor (*CASR*), a G protein coupled‐receptor, is epigenetically silenced in cancers, and therefore we examined its role in GEP‐NET subtypes. Using RNA‐Scope and quantitative PCR analyses in two independent tumour cohorts from Europe (*n* = 18 patients) and the USA (*n* = 46 patients) we showed that *CASR* mRNA is almost completely absent in gastrinomas, insulinomas and non‐functioning pancreatic NETs. Furthermore, immunohistochemical staining confirmed a significant reduction in CaSR protein expression in all GEP‐NET subtypes, compared to normal islets. DNA methylationEPIC and ATAC‐seq analyses in the pancreatic NET cell line QGP‐1 showed the CaSR promoter was both hypermethylated and in a region of closed chromatin. Furthermore, transfection of wild type CaSR into QGP‐1 cells decreased cell viability, in keeping with the CaSR having a role in cellular proliferation. In summary, our study reveals that CaSR expression is decreased in GEP‐NETs and that this reduced expression is likely due to DNA methylation and chromatin changes. Moreover, we demonstrate that transfection of the CaSR into a PNET cell line reduces cell viability, thereby indicating that the CaSR acts as a tumour suppressor in this tumour type.

## What's New?

Epigenetic regulation has been suggested to play a major role in the pathogenesis of gastroenteropancreatic neuroendocrine tumours, but the underlying mechanisms remain poorly understood. Here, the authors show in two independent cohorts that the calcium‐sensing receptor (CaSR) is down‐regulated in gastroenteropancreatic neuroendocrine tumours and that this reduced expression is likely due to alterations in DNA methylation and chromatin organisation. Moreover, they show that transfection of gastroenteropancreatic neuroendocrine tumour cell lines with CaSR decreases cell viability. The findings shed light on the specific role of CaSR as a tumour suppressor in gastroenteropancreatic neuroendocrine tumours.

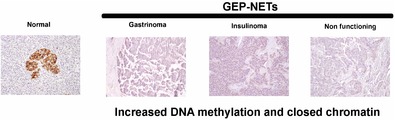

## 1 INTRODUCTION

Gastroenteropancreatic neuroendocrine tumours (GEP‐NETs) are relatively rare, with a worldwide incidence of 0.3–0.6 per 100,000 people, but they can cause severe morbidity due to hormone hypersecretion and have a poor prognosis with a median overall survival of 75–124 months.^1^ GEP‐NETs may be functional and secrete hormones, for example, insulinomas and gastrinomas, or be non‐functional, that is, non‐secreting.^2^ GEP‐NETs can occur sporadically or as part of a hereditary syndrome, such as multiple endocrine neoplasia type 1 (MEN1), or Von Hippel–Lindau syndrome, which are caused by mutations in the specific genes *MEN1* and *VHL*, respectively.^2^ Although hereditary syndromes have provided insights into the genetic drivers of these NETs, large‐scale sequencing analysis of GEP‐NETs, particularly pancreatic NETs (PNETs), has revealed remarkably few additionally mutated genes, compared to the number of mutated genes observed in other tumour types.^3^ Thus, the most commonly mutated genes in sporadic GEP‐NETs are *MEN1*, death‐domain‐associated protein (*DAXX*), and ATRX Chromatin Remodeler (*ATRX*), occurring in 40%, 25%, and 17% of tumours, respectively.^4^, ^5^ All three of these genes are well‐described tumour suppressors that are also known to play a role in other cancers. Menin, encoded by *MEN1*, is a member of multiple complexes that can regulate gene transcription and proliferation, for example, it is known to form part of the complexes responsible for the modification of histone tails, including histone deacetylase complexes that can remove acetyl residues from histone tails, and histone demethylase complexes (KDM) that can remove methyl groups from histone tails.^2^ *DAXX* and *ATRX* form the histone 3.3 (H3.3) chaperone complex, which loads H3.3 into the telomeric region of the chromatin that protects the DNA from alternate lengthening of telomers, a mechanism commonly used by cancer cells.^6^ Menin, DAXX, and ATRX are therefore key proteins involved in the maintenance of gene transcription, and therefore their loss of function is associated with many cancer types for example leukaemia, prostate cancer, gliomas, and sarcomas.^2^, ^6^, ^7^, ^8^ Mutations in other genes in sporadic GEP‐NETs, such as phosphatase and tensin homolog (*PTEN*), and those in the mammalian target of rapamycin (mTOR) signalling pathway have also been identified but at lower frequencies.^4^, ^5^ Considering the relatively low mutational burden observed in these tumours, and the observation that the three most commonly mutated genes, *MEN1*, *DAXX*, and *ATRX* play a role in histone regulation, it is hypothesised that epigenetic regulation of gene expression may play a major role in GEP‐NET tumorigenesis. Therefore, it is likely that mutational analysis alone will not be sufficient to gain full insight into GEP‐NET development.

The calcium‐sensing receptor (CaSR) is a G protein‐coupled receptor^9^ that is predominantly expressed in the parathyroid glands, kidneys, and bone and plays an important role in calcium homeostasis, whereby it senses extracellular calcium concentrations and controls intracellular signalling pathways, including intracellular calcium release, to modulate cellular functions including parathyroid hormone (PTH) secretion and renal tubular calcium handling.^9^ In addition, the CaSR has non‐calcitropic roles and has been shown to regulate gene expression, cellular proliferation, differentiation and apoptosis, glucose metabolism, and enteroendocrine hormone secretion.^9^ Furthermore, depending on the cellular context, the CaSR has been reported to act as both an oncogene and tumour suppressor. For example, in breast cancer, the CaSR is upregulated increases proliferation and migration, and inhibits apoptosis whilst in prostate cancer higher CaSR expression is linked with increased mortality where it is thought to utilise dietary calcium to promote cell proliferation, invasion, and tissue inflammation.^10^, ^11^, ^12^

In contrast, the CaSR is reported to be epigenetically silenced in several cancers, as described below, and we have previously shown that the epigenetic targeting compound JQ1, which inhibits the bromo and extra terminal domain family of proteins that recognise acetylated histone residues and promote gene transcription, down‐regulates the CaSR in pituitary adenoma cells.^13^ Thus, CaSR expression has been reported to be decreased in neural tumours and colorectal cancer due to DNA methylation and histone modification, and in turn, causes a reduction in cellular proliferation.^14^, ^15^ In addition, the CaSR is also expressed in the gastrointestinal tract and pancreatic islets where it has a role in regulating digestion glucose metabolism, and insulin and glucagon secretion, respectively.^9^ However, the role of the CaSR in GEP‐NETs tumorigenesis is unknown. We hypothesised that CaSR expression may be altered in GEP‐NETs as a result of epigenetic changes and investigated this hypothesis by examining CaSR expression in insulinomas, gastrinomas, and non‐functioning PNETs (NF‐PNETs), as well as methylation and chromatin state of the *CASR* gene locus in PNET cell lines.

## 2 MATERIALS AND METHODS

### 2.1 Patient information and sample details

Formalin‐fixed paraffin‐embedded (FFPE) samples of *n* = 18 GEP‐NETs (*n* = 6 insulinomas, *n* = 6 NF‐PNETs and *n* = 6 gastrinomas) were obtained from the Medical University of Vienna (Table 1). All insulinomas were grade 1 with a mean diameter size of 13.3 mm. The mean patient age was 71 years, and 67% were female. NF‐PNET tumour grades ranged from 1 to 3 with a mean tumour diameter of 56 mm, the mean patient age was 54 years, and 33% were female. Gastrinoma tumour grade ranged from 1 to 3 with a mean tumour diameter of 31 mm, the mean patient age was 54 years, and 17% were female. Fresh normal pancreatic tissue (consisting of exocrine and endocrine cells) was obtained from the Oxford Radcliffe Biobank from *n* = 5 patients undergoing pancreatic surgery for non‐NET related conditions, which were either fixed in 4% paraformaldehyde for 48 h before being embedded in paraffin or frozen at −80°C in freezing media (RPMI media containing 10% DMSO). Individuals had a mean age of 61 years, and 40% were female (Table 1). RNA from *n* = 46 GEP‐NETs was obtained from the Mayo Clinic (Rochester) and included 14 insulinomas (age range 37–73 years; grade 1–2), 25 NF‐PNETs (age range 20–78 years, grade 1–3), and 7 gastrinomas (age range 48–64 years, grade 1–2) (Table 1).

**TABLE 1:** Clinical details of formalin fixed paraffin embedded tissue samples obtained from Medical University of Vienna and Oxford Radcliffe Biobank, and fresh frozen samples obtained from the Mayo clinic.

Samples	Identifier	Gender (F/M)	Age (years)	NET grade	Tumour size (mm)	ENETs stage
Vienna						
Insulinoma (*n* = 6)	INSa^a^	M	62	1	5	1
	INSb	F	74	1	15	1
	INSc^a^	M	80	1	8	1
	INSd^a^	F	94	1	17	1
	INSe^a^	F	43	1	20	1
	INSf	F	72	NR	15	1
Mean (sd) or proportion (%)		4/6 (67%; F)	70.8 (17.2)	G1 = 5	13.3 (5.7)	E1 = 1
Non‐functioning PNET (*n* = 6)	NFa	M	71	1	60	3
	NFb	M	58	2	27	2
	NFc^a^	F	40	3	180	3
	NFd^a^	M	68	1	17	1
	NFe^a^	M	34	2	30	2
	NFf^a^	F	55	2	22	2
Mean (sd) or proportion (%)		2/6 (33%; F)	54.3 (14.8)	G1 = 2, G2 = 3, G3 = 1	56 (62.6)	E1 = 1, E2 = 3, E3 = 2
Gastrinoma (*n* = 6)	GSa	M	55	1	70	4
	GSb	M	77	1	10	1
	GSc	M	67	1	11	2
	GSd	M	42	1	40	3
	GSe	F	61	3	42	3
	GSf	M	22	2	11	2
Mean (sd) or proportion (%)		1/6 (17%; F)	54 (19.6)	G1 = 4, G2 = 1, G3 = 1	30.6 (24.3)	E1 = 1, E2 = 2, E3 = 2, E4 = 1
Oxford Biobank						
Normal pancreas	Pa	M	50	NA	NA	NA
	Pb	F	63	NA	NA	NA
	Pc	F	71	NA	NA	NA
	Pd	M	55	NA	NA	NA
	Pe	M	68	NA	NA	NA
Mean (sd) or proportion (%)		2/5 (40%; F)	61.4 (8.8)			
Mayo Cinic						
Insulinoma (*n* = 14)	1	F	60	1	NR	NR
	2	M	63	1	NR	NR
	3	F	54	1	NR	NR
	4	M	73	1	NR	NR
	5	M	57	1	NR	NR
	6	F	54	1	NR	NR
	7	F	50	1	NR	NR
	8	M	59	1	NR	NR
	9	F	39	1	NR	NR
	10	M	72	2	NR	NR
	11	F	37	2	NR	NR
	12	M	53	2	NR	NR
	13	M	73	1	NR	NR
	14	F	69	1	NR	NR
Mean (sd) or proportion (%)		7/14 (50%; F)	58.1 (11.5)	G1 = 11, G2 = 3		
Non‐functioning PNET (*n* = 25)	1	F	57	1	NR	NR
	2	F	74	1	NR	NR
	3	M	70	1	NR	NR
	4	M	78	1	NR	NR
	5	F	72	1	NR	NR
	6	F	61	2	NR	NR
	7	F	63	2	NR	NR
	8	F	20	2	NR	NR
	9	F	64	2	NR	NR
	10	F	56	2	NR	NR
	11	M	76	2	NR	NR
	12	F	57	2	NR	NR
	13	F	61	2	NR	NR
	14	M	73	3	NR	NR
	15	M	72	3	NR	NR
	16	F	50	3	NR	NR
	17	F	64	1	NR	NR
	18	F	66	1	NR	NR
	19	F	39	1	NR	NR
	20	M	33	2	NR	NR
	21	M	61	1	NR	NR
	22	M	66	2	NR	NR
	23	F	68	2	NR	NR
	24	F	51	1	NR	NR
	25	M	57	2	NR	NR
Mean (sd) or proportion (%)		16/25 (64%; F)	58.7 (13.7)	G1 = 10, G2 = 12, G3 = 3		
Gastrinoma (*n* = 7)	1	M	48	1	NR	NR
	2	M	56	1	NR	NR
	3	M	49	1	NR	NR
	4	M	60	1	NR	NR
	5	F	50	2	NR	NR
	6	M	64	NR	NR	NR
	7	F	53	NR	NR	NR
Mean (sd) or proportion (%)		5/7 (71.4; F)	58.7 (6.0)	G1 = 4, G2 = 1		

Abbreviations: NR, not recorded; NA, not applicable.

^a^ Neuroendocrine tumours where CASR expression of adjacent normal islets were also analysed.

### 2.2 RNA scope and immunohistochemistry

FFPE tissue was obtained following surgical resection, and serial sections of 5 μm cut. Serial tissue sections were dewaxed and hydrated before RNA‐scope and immunohistochemistry analysis. RNA‐scope was performed using the RNA‐Scope 2.5 HD Detection Kit‐RED, with a HybEZ Hybridisation System (both Advance Cell Diagnostics), and standard pre‐treatment and hybridisation conditions, using probes specific for the CaSR and according to the manufacturer's instructions, as previously described.^16^ Sections were viewed by light microscopy using an Eclipse E400 microscope (Nikon), utilising a DXM1200C digital camera, and each individual red dot was counted using NIS‐Elements BR software (both Nikon). Haematoxylin and eosin (H&E) staining was performed as previously described.^16^ For immunohistochemistry, antigen retrieval was performed at 120°C in a citrate buffer solution (pH 6); blocking in 10% donkey serum; incubation with primary antibody for CaSR (5C10, ADD, ThermoFisher Scientific), or synaptophysin (AB 32127, AbCam) followed by mouse or rabbit HRP‐conjugated secondary antibody (Jackson Laboratories); and visualisation using the diaminobenzidine (DAB)‐kit (Dako), as previously described.^17^ Sections were viewed by light microscopy using an Eclipse E400 microscope (Nikon), equipped with a DXM1200C digital camera. Staining was quantified by generating an H score in QuPath software.^18^

### 2.3 Cell culture and transfections

HEK293T human embryonic kidney cells (ATCC #CRL‐3216; RRID CVCL_0063) and QGP‐1 human pancreatic neuroendocrine tumour cells (JCRB Cell Bank #JCRB0183; RRID CVCL_3143) were cultured in high glucose DMEM, and RPMI media (Gibco), respectively, supplemented with 10% fetal calf serum (Sigma‐Aldrich), maintained at 37°C, 5% (vol/vol) CO_2_, and tested for mycoplasma using the MycoAlert kit (Lonza, Basel, Switzerland). All experiments were performed with mycoplasma‐free cells, and all cell lines have been authenticated using STR profiling within the last 3 years. Cells were split at least twice per week with HEK293T and QGP‐1 cells having a doubling time of 24 and 48 h, respectively. Wild‐type CaSR pFLAG‐N1‐*CASR* and pCMV6‐Entry‐*DCC*‐Myc‐DDK (Origene) constructs were transfected into HEK293T and QGP‐1 cells using Lipofectamine 2000 (Life Technologies) as previously described.^19^

### 2.4 Methylation array

DNA was extracted from QGP‐1 cells using the Puregene kit (Qiagen), and DNA integrity and concentration were assessed using an Agilent 2100 Tapestation. A total of 1 μg of DNA was bisulfite‐only converted (TrueMethyl oxBS‐Seq Module) and sent for MethylationEPIC array analysis (Cambridge Genomic Services). Approximately 850,000 CpG sites were interrogated across the genome and raw beta values for each CpG site were reported and generated in R^20^ using the ChAMP pipeline.^18^ Beta values (range: 0–1) for each CpG site were determined after loading the raw data files into R (champ.load) and then normalising each CpG site (champ.norm). Unmethylated CpG sites were defined as having a beta value <0.3, and methylated CpG sites had a beta value >0.7. Regions of unmethylated DNA in normal pancreatic alpha (*α*), beta (*β*), and delta (*δ*) cells, are, publicly available.^21^ This epigenetic data was then matched to CpG sites interrogated in the methylationEPIC array using genomic coordinates and identifiers (e.g., gene ID) in R. Matched methylation data from QGP‐1 cells and normal islets (*α*, *β*, and *δ* cells) were extracted for three genes: *CASR*, Somatostatin receptor 3 (*SSTR3*) and glycerylaldehyde‐3‐phosphatase dehydrogenase (*GAPDH*). CpG islands, transcriptional start sites, and their gene locations were identified using the UCSC Genome Browser (GRCh37/hg19). Quality statistics for the methylation array data are included in Supplementary Table 1.

### 2.5 Assay for transposase‐accessible chromatin using sequencing (ATAC‐seq)

Freshly cultured QGP‐1 cells were pelleted, washed in PBS, and nuclei extracted using the Chromium Nuclei Isolation Kit (10X Genomics). Nuclei target recovery was 6000, and three single nuclear (sn)ATAC‐seq libraries were generated using the chromium NET GEM single‐cell ATAC kit (10X Genomics). The three libraries were sequenced on an Illumina NextSeq500 instrument and counted and aggregated with depth normalisation with cellranger‐atacv1.2 (10xGenomics, GRCh38/hg38) and sequenced with a configuration of 150 bp paired‐end reads. A median number of 15,546 fragments per cell were captured in an estimated total of 5129 cells, post normalisation a total of 1.05E+08 fragments were mapped. Datasets were processed using Seurat v4.3.0 and Signac 1.9.0 in R. Quality control was undertaken by removing low‐quality cells to leave cells with peak region fragments >3000 and < 50,000, percentage of reads in peaks >30, blacklist ratio <0.0025, nucleosome signal <4, and transcription start site enrichment score of >2. Normalisation was performed using term‐frequency inverse‐document frequency (TFIDF), and dimensional reduction with singular value decomposition of the TFIDF matrix using all features to generate latent semantic indexing (LSI). The first LSI component was removed from downstream analysis due to a high correlation between this LSI component and the total number of cell counts indicating that it was capturing technical rather than biological variation. Clustering was undertaken using UMAP, and ‘FindNeighbours’ and ‘FindClusters’ functions in Seuratv4.3.0. A gene activity matrix was generated via the ‘GeneActivity’ function in Seurat which extracts gene coordinates, extends them to include the 2 kb upstream region, and counts the number of fragments mapping to regions; this activity matrix was log‐normalised and clusters inspected for markers of apoptosis (BCL2 apoptosis regulator (*BLC2*), BCL2 associated apoptosis regulator (*BAX*), and tumour protein P53 (*TP53*)). These markers were homogenously distributed between clusters; thus, clusters were combined into one group and all remaining QGP‐1 cells (3313 cells with 139,600 features) were considered in downstream analyses. snATAC‐seq narrow peaks were defined using the ‘CallPeaks’ function in Signac which utilises Models‐based analysis of ChIP‐Seq (MACS2). The *CASR* locus was examined for open chromatin peaks, and compared to the genomic locus of the *SSTR3* gene which is not expressed and expected to be in a region of closed chromatin, and *GAPDH* which is highly expressed and therefore expected to be in a region of open chromatin.^22^ Quality statistics for the ATAC‐Seq data are included in Supplementary Table 2.

### 2.6 In vitro assays

Cell viability was assessed using the CellTiter Blue Cell Viability assay (Promega, Southampton, UK), at 48 h and 7 days post‐transfection, whereby 20 μL of CellTiter Blue reagent was added per well, incubated for 2 h at 37°C, 5% (vol/vol) CO_2_, and the fluorescent outputs were read on a PHERAstar microplate reader (BMG Labtech, Aylesbury, UK) at 530 nm excitation and 580 nm emission. The reading at 48 h was subtracted from the reading at 7 days and data represented relative to untransfected cells. Of note, due to the length of the assay, no additional calcium was added to the cell culture media prior to assessing cell proliferation. The assay was performed in *n* = 4 biological replicates. For measurement of intracellular calcium responses at 48 h post transfection, cells were washed with 100 μL Complete Imaging Buffer (150 mM NaCl, 2.6 mM KCl, 1.18 mM MgCl_2_,10 mM HEPES, 0.1 mM CaCl2, pH 7.4), loaded with Fluo‐4 dye (Complete Imaging Buffer supplemented with; 1 μM Fluo‐4 AM, 0.01% pluronic F‐127, 0.5% BSA), and incubated for 60 min at 37°C. Cells were washed again before the addition of a further 100 μL of Complete Imaging Buffer and incubated for 30 min at room temperature in the dark. Using an automated system, calcium chloride was injected into the wells to achieve extracellular calcium concentrations ranging from 0.05 to 2 mM. Control wells were injected with 10 μM ionomycin. Changes in intracellular calcium concentrations were recorded via detection of fluorescence for 30 s using a PHERAstar microplate reader (BMG Labtech) at 37°C with an excitation filter of 485 nm and an emission filter of 520 nm. The peak mean fluorescence ratio of the transient response following each individual stimulus was measured using MARS data analysis software (BMG Labtech). Relative fluorescence units were normalised to the fluorescence stimulated by ionomycin to account for differences in cell number and loading efficiency. Assays were performed using six biological replicates (independently transfected wells, performed on at least six different days). Nonlinear regression of concentration‐response curves was performed using GraphPad Prism to determine the maximal response.

### 2.7 Western blot analysis

QGP‐1 cells were lysed in NP40 lysis buffer and prepared in 4x Laemmli loading dye (BioRad), resolved using 6% or 10% SDS‐PAGE gel electrophoresis, and transferred to polyvinylidene difluoride membrane, as described previously.^23^ Membranes were probed with the primary antibodies mouse‐anti CaSR (5C10, ADD, ThermoFisher Scientific) and rabbit anti‐DCC (Abcam, ab273570), or rabbit‐anti calnexin (Millipore, Ab2301), and anti‐rabbit or anti‐mouse horseradish peroxidase‐conjugated secondary antibody (Santa Cruz Biotechnology), with blots visualised using Pierce Enhanced chemiluminescence Western blotting substrate (Thermo Fisher Scientific), as described in Reference [23]. Calnexin protein expression was used as a loading control. Densitometry analysis was performed by calculating the number of pixels per band using ImageJ software. Data are represented as the number of pixels in the protein band, relative to the number of pixels in the corresponding calnexin band.

### 2.8 Quantitative reverse transcription PCR (qRT‐PCR)

Total RNA was extracted from frozen tissue using the RNeasy Mini kit (Qiagen), and 1 μg used to generate cDNA using the Quantitect Reverse Transcription kit (Qiagen). Quantitect primers (Qiagen) for *CASR*, as well as the housekeeper genes *GAPDH*, calnexin (*CANX*), and beta‐2‐microglobulin were used with the Quantitect SYBR green kit (Qiagen), on a RotorGene 5, as previously described.^23^ Each test sample was normalised to the geometric mean of the housekeeper genes. The relative expression of the target cDNA in all qRT‐PCR studies was determined using the Pfaffl method.^24^

### 2.9 Statistical analysis

All statistical comparisons were performed using GraphPad Prism version 9. One‐way ANOVA comparisons were used for RNA‐Scope, immunohistochemistry, qRT‐PCR, western blotting, and proliferation analyses. For intracellular calcium assays, a two‐way ANOVA with Tukey's multiple comparison test was performed.

## 3 RESULTS

### 3.1 CaSR expression is down‐regulated in GEP‐NETs

**FIGURE 1:**
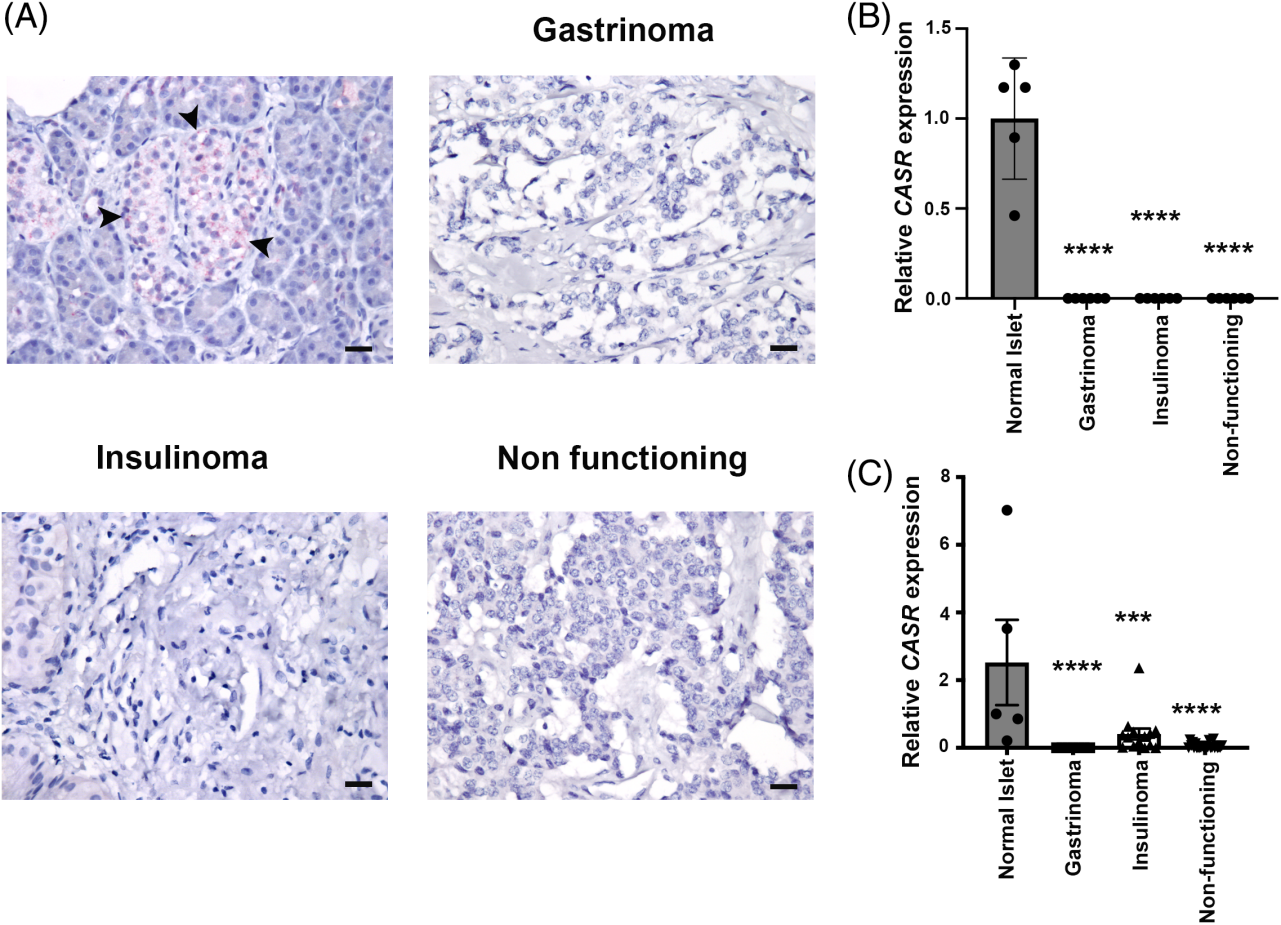
Analysis of *CASR* mRNA expression in GEP‐NETs. *CASR* mRNA expression was examined in FFPE sections of normal pancreatic islets, gastrinomas, insulinomas and NF‐PNETs using RNA scope (A). Positive *CASR* staining is shown by red dots, and the scale bar represents 10 μm. The intensity of staining which consisted of the total number of red dots was quantified from six insulinomas, six gastrinomas and six NF‐PNETs, and represented relative to the average value of five normal islets (B). Statistical significance was determined using one‐way ANOVA and compared to normal islets, ****p < .0001. *CASR* RNA expression was also examined in an independent cohort of fresh frozen insulinomas (*n* = 14), gastrinomas (*n* = 7) and NF‐PNETs (*n* = 25), which were compared to normal islets (*n* = 5) (C). Data are represented relative to the average score for normal islets, statistical significance was determined by one‐way ANOVA with each subtype compared to the normal islet group ***p < .005, ****p < .0001.

**FIGURE 2:**
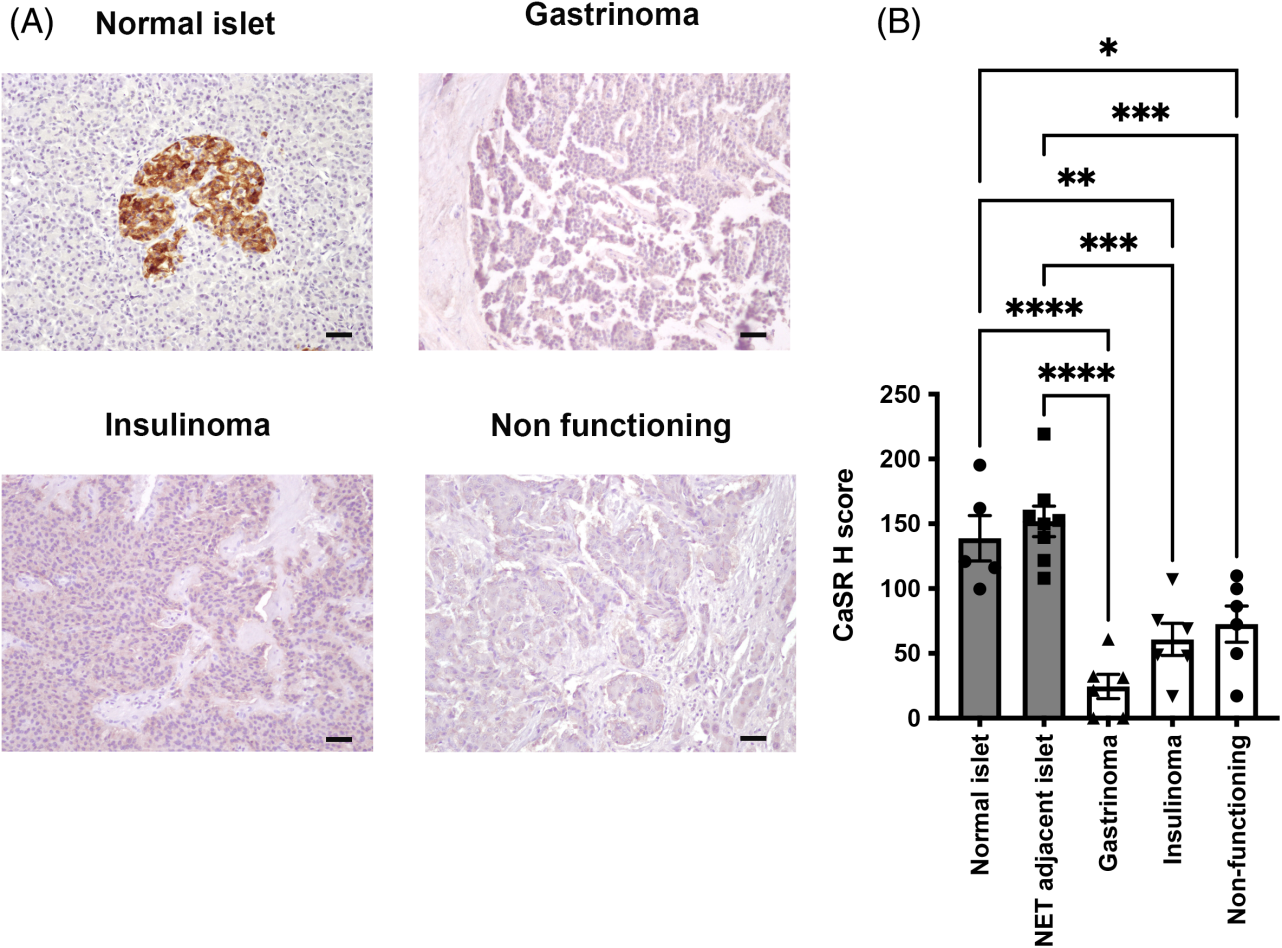
Analysis of CaSR mRNA in GEP‐NETs. CaSR expression was examined in FFPE sections of normal pancreatic islets, gastrinomas, insulinomas, and NF‐PNETs using immunohistochemistry (A). Positive staining is shown in brown, the scale bar represents 50 μm. (A) CaSR H score was also generated by quantifying the staining intensity using QPath software in five normal islets, eight normal adjacent islets to GEP‐NETs, six insulinomas, six gastrinomas and six NF‐PNETs (B). Statistical significance was determined using one‐way ANOVA corrected for multiple comparisons with significance reported relative to normal islets, and normal adjacent islets *p < .05, **p < .005, ***p < .0005, ****p < .0001.

To determine whether CaSR expression is altered in GEP‐NETs, we examined RNA and protein levels in a cohort of FFPE tissues from the Medical University of Vienna, consisting of six normal pancreatic tissue samples, six gastrinomas, six insulinomas, and six NF‐PNETs. In addition, RNA expression was assessed in an independent cohort of 5 normal pancreata (obtained from the Oxford Racliffe Biobank, UK) and 46 (7 gastrinomas, 14 insulinomas, and 25 NF‐PNETs) fresh frozen GEP‐NET samples obtained from the Mayo Clinic, USA (Table 1). All samples were stained with H&E, and immunostained with the neuroendocrine tissue marker synaptophysin to confirm that the tumours were NETs (Supplementary Figure 1). RNA‐Scope analysis indicated that *CASR* mRNA was reduced in all GEP‐NET tissue types but present in normal islets (Figure 1A). Quantification demonstrated that *CASR* mRNA expression was significantly higher in normal islets (mean relative expression = 1, range = 0.46–1.30) when compared to gastrinomas, insulinomas, and NF‐PNETs (mean relative expression = 0 and *p* < .0001 for all tumour subtypes, Figure 1B **)**. This reduction in *CASR* mRNA was confirmed by quantitative PCR in the independent cohort of fresh frozen tissue from the Mayo Clinic, which revealed significantly reduced *CASR* expression in gastrinomas, insulinomas, and NF‐PNETs compared to that in normal islets (*p* < .00001, *p* < .0005, and *p* < .0001, respectively, Figure 1C). Of note, although the pancreatic exocrine tissue is negative for CaSR expression, islets were not isolated for the quantitative PCR which may cause variations in the fold changes seen compared to the RNA Scope data. CaSR protein expression was examined using immunohistochemical analysis, and this demonstrated that CaSR expression is high in normal islets, but that there is little to no expression in exocrine tissue (Figure 2A). Normal islets adjacent to the NETs could be identified in eight patients (four insulinomas and four NF‐PNETs), and as with the non‐adjacent normal islets highly expressed CaSR. To quantify differences in CaSR expression, H scores for each sample were generated using QPath software, and when compared to normal islets, and normal adjacent islets, a significant reduction in CaSR expression was observed in gastrinomas (5.75‐fold, and 6.3‐fold, both *p* < .0001), insulinomas (2.3‐fold *p* < .005, and 2.5‐fold *p* < .005), and NF‐PNETs (1.9‐fold *p* < .05 and 2‐fold *p* < .005) (Figure 2A, B), consistent with the RNA results.

### 3.2 The CASR locus is hypermethylated and in a region of closed chromatin in QGP‐1 cells

**FIGURE 3:**
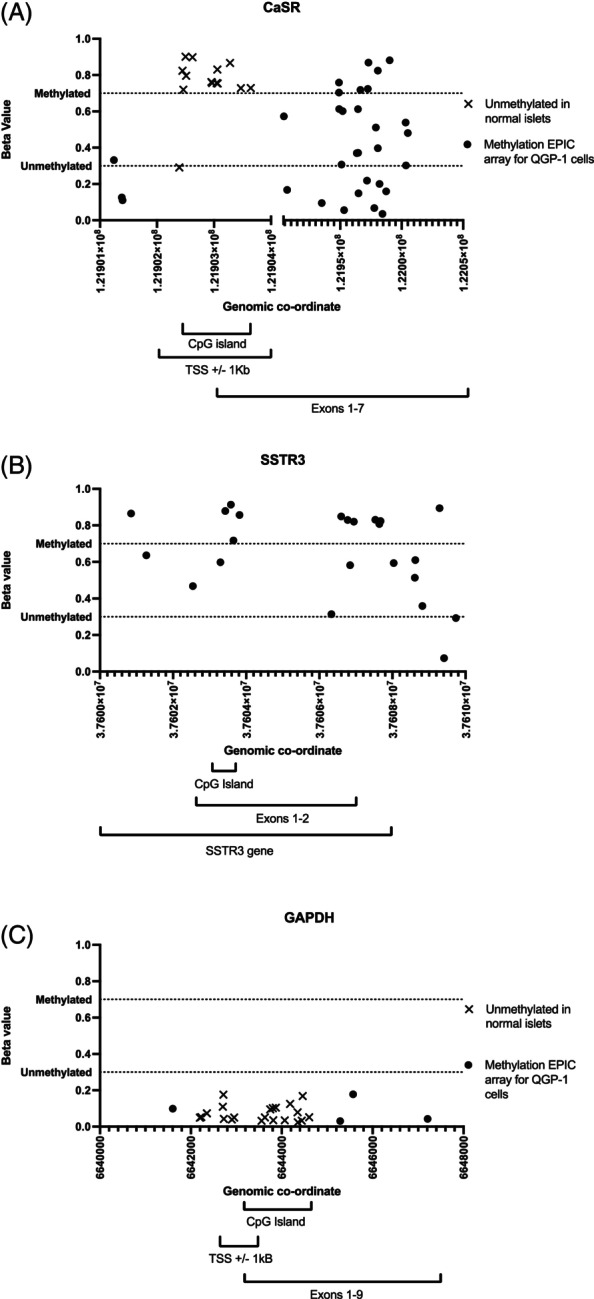
Analysis of *CASR* DNA methylation. CpG sites for *CASR*, *SSTR3* and *GAPDH* were interrogated in QGP‐1 cells using the methylationEPIC array and compared to publicly available methylation data for normal pancreatic *α*, *β*, and *δ* cells. In total, 45 CpG sites were interrogated for the *CASR* (A), 23 CpG sites were interrogated for *SSTR3* (B), and 25 CpG sites were interrogated for *GAPDH* (C). Each data point represents the beta value for a specific CpG site in QGP‐1 cells (*y*‐axis) plotted against the genomic location (*x*‐axis). The dotted lines indicate the thresholds for a CpG site being classed as methylated (beta value >0.7) or unmethylated (beta value <0.3). The genomic locations for the coding region, CpG island and TSS ±1 kb for each gene were obtained from UCSC Genome Browser. CpG sites unmethylated in *α*, *β*, and *δ* cells are shown as crosses, CpG sites unmethylated in *β* cells only are shown as an asterisk and CpG sites unmethylated in *α* cells only are shown as a star. The remaining CpG sites represented as black circles were not within unmethylated regions in normal *α*, *β*, and *δ* cells. There were no sites in which only QGP‐1 and *δ* cells were unmethylated.

We hypothesised that CaSR expression may be reduced in GEP‐NETs due to epigenetic changes or alterations in chromatin accessibility. To investigate these hypotheses, we first undertook global DNA methylation analysis in QGP‐1 cells using an EPIC array to determine which gene transcriptional start sites (TSS) were methylated, and therefore likely to show reduced transcription. QGP‐1 cells were used as they are a cell line isolated from an NF‐PNET, and have been described as biologically representative of immature pancreatic *β*/*δ* cells. As the exact cell type of origin of the QGP‐1 cells is not known their methylation profiles were compared to publicly available data of normal pancreatic *α*, *β*, and *δ* cells.^21^, ^25^ The methylationEPIC array interrogated 45, 23, and 25 CpG sites in QGP‐1 cells for the *CASR*, *SSTR3*, and *GAPDH* genes, respectively (Figure 3A–C). In normal pancreatic *α*, *β*, and *δ* cells, unmethylated regions of 704, 678, and 544 base pairs located ± 1 kb from the TSS for the CaSR, respectively, were identified. For the *CASR* there were 14 CpG sites assessed within ±1 kb of the TSS, with 50% (7/14), 71% (10/14), 43% (6/14), and 7% (1/14) CpG sites unmethylated in normal *α*, *β*, *δ* and QGP‐1 cells, respectively. All CpG sites unmethylated in normal pancreatic islets (*α*, *β*, and *δ* cells) were methylated in QGP‐1 cells (beta value >0.7). All normal islet cell subtypes had a shared region of unmethylated DNA 544 bp long at the TSS of the *CASR* (hg19 location: 121902612‐121903156; Figure 3A). *SSTR3*, a gene not reported to be expressed in QGP‐1 cells and therefore acts as a negative control for transcription, was also investigated. All *SSTR3* CpG sites were methylated in the normal *α*, *β*, and *δ* cells, and QGP‐1 cells (Figure 3B). In contrast, for *GAPDH*, a housekeeper gene highly expressed in most cell types, all CpG sites were unmethylated in normal pancreatic tissue (*α*, *β*, and *δ*) and QGP‐1 cells (Figure 3C).

**FIGURE 4:**
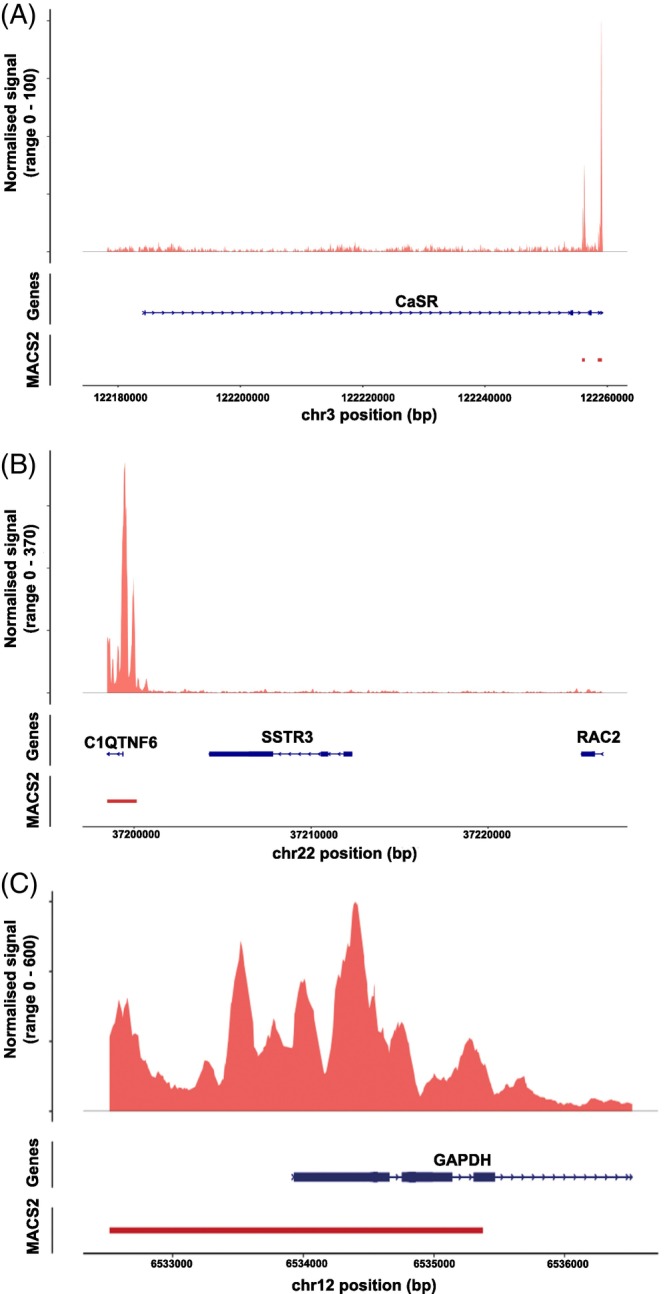
Analysis of chromatin structure at the *CASR* locus. ATAC‐Seq was undertaken in QGP‐1 cells to determine whether alterations in chromatin accessibility at the *CASR* locus may be responsible for the observed reduction in CaSR expression. Normalised chromatin accessibility signal is represented in the upper panel, and areas of open chromatin identified via MACS2 denoted in lower panels (A). Somatostatin receptor 3 (*SSTR3*) which is not expressed in QGP‐1 cells was used as a negative control for open chromatin (B), and *GAPDH* which is known to be expressed in QGP‐1 cells, was used as a positive control for open chromatin (C). Gene locations are shown in blue with boxes denoting the exons, and arrows indicating the direction of transcription.

Areas of open chromatin in QGP‐1 cells were also assessed using snATAC‐seq and identified using MACS2. No areas of open chromatin were observed in proximity to the CaSR TSS, and the closest region of open chromatin was ~71 kb downstream of the TSS (Figure 4A). *SSTR3* chromatin was also closed in regions in proximity to the TSS of *SSTR3* with the closest region of open chromatin ~12 kb downstream of the TSS (Figure 4B). In contrast, chromatin was open in the region of the *GAPDH* TSS (Figure 4C). These findings suggest that reduced CaSR expression in GEP‐NETS may arise due to increased DNA methylation and reduced chromatin accessibility.

### 3.3 QGP‐1 cells transfected with CaSR respond to calcium and have reduced viability

**FIGURE 5:**
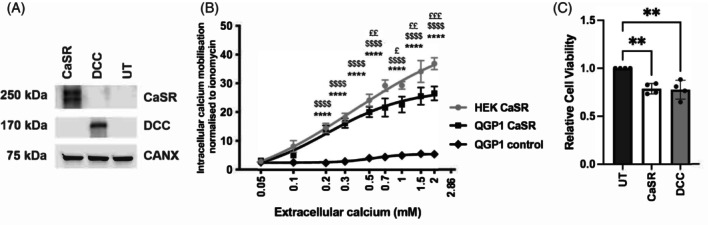
Assessment of CaSR function after its reintroduction into QGP‐1 cells. To determine if the CaSR functions in GEP‐NETs we transfected wild type CaSR into the NF‐PNET cell line QGP‐1. In addition, we also transfected QGP‐1 cells with the known tumour suppressor DCC, as a comparator. Expression of CaSR and DCC was confirmed by Western blot analysis (A). Calnexin (CANX) was used as a loading control. Intracellular calcium mobilisations assays were performed to determine if the re‐expressed CaSR could increase intracellular calcium in response to treatment with increasing extracellular calcium concentrations (B). QGP‐1 cells were treated with 0–2 mM calcium and intracellular calcium response is represented relative to ionomycin response. Untransfected QGP‐1 cells were used as a negative control (QGP‐1 control), and HEK293T cells transfected with the CaSR were used as a positive control (HEK CaSR). Statistical significance from *n* = 6 biological replicates was determined by two‐way ANOVA with * = QGP‐1 control versus QGP‐1 CaSR, $ = QGP‐1 control versus HEK CaSR and £ = QGP‐1 CaSR versus HEK CaSR; £ < 0.05, ££ < 0.005, £££ < 0.0005, ****/$$$$/££££ < 0.0001. The effect of CaSR expression on cell viability was also assessed using Cell Titer Blue assay (C). Viability of QGP‐1 cells transfected with CaSR or DCC was assessed after 96 h, and untransfected cells (UT) were used as a control. Data are represented relative to UT cells. Statistical analysis relative to UT on *n* = 4 biological replicates was assessed by one‐way ANOVA **p < .005.

To determine the likely function of the CaSR in GEP‐NETs, and whether its reduced expression may contribute to tumuorigenesis, we expressed the CaSR in QGP‐1 cells. Lack of endogenous CaSR expression, as well as expression of transfected CaSR, was confirmed by Western blot analysis, and Calnexin was used as a housekeeper (Figure 5A). To ensure that the transfected CaSR was functional, we undertook intracellular calcium assays which showed that transfected CaSR could significantly increase intracellular calcium mobilisation in response to treatment with 0.2, 0.3, 0.5, 0.7, 1, 1.5 and 2 mM extracellular calcium, compared to untransfected cells (*p* > .001, Figure 5B). This response was similar to intracellular calcium mobilisation assays we undertook in HEK293T cells transfected with the *CASR* (Figure 5B); HEK293T cells have been widely utilised to investigate CaSR function and therefore used as a positive control.^26^, ^27^, ^28^ The CaSR has been shown to contribute to cellular proliferation in some cell types, and we therefore examined the viability of transfected cells. QGP‐1 cells transfected with a CaSR‐expression construct showed significantly reduced viability compared to untreated cells (1.4‐fold, *p* < .005, Figure 5C), however, there was no alteration in the viability of HEK293T cells transfected with a CaSR‐expression construct (Supplementary Figure 2). As a positive control, QGP‐1 cells were also transfected with the known tumour suppressor protein Deleted in colorectal cancer (*DCC*) that encodes the netrin receptor which represses tumour formation when not activated by its ligand netrin.^29^ As expected, *DCC* transfected QGP‐1 cells displayed reduced viability (1.4‐fold, *p* < .005, Figure 5C **)**. Expression of DCC in QGP‐1 cells was confirmed by western blot (Figure 5A).

## 4 DISCUSSION

Our study reveals that CaSR expression is significantly reduced in gastrinomas, insulinomas, and NF‐PNETs and that the *CASR* locus is highly methylated with closed chromatin formation in the NET cell line QGP‐1. Furthermore, we show that when the CaSR is introduced into an NF‐PNET cell line, not only was the CaSR able to increase intracellular calcium concentrations, but it also significantly reduced cell viability, consistent with evidence that the CaSR may play a tumour suppressor role in certain tissues.

Our data indicates that the CaSR is down‐regulated in GEP‐NETs and highlights the importance of a hypothesis‐driven approach for identifying potential aberrant protein expression and signalling in this tumour type, especially as the underlying biology of the tumorigenic process is still not well understood. Currently, there are several studies reporting DNA mutations that contribute to GEP‐NET development, the key drivers of which include the chromatin modifiers *MEN1* and *DAXX/ATRX*, as well as studies investigating the DNA methylome which have highlighted genes including *RASSF1* and *CTNNB1* to be silenced through promoter hypermethylation.^4^, ^5^, ^30^ This indicates the importance of epigenetic mechanisms in NETs, however, the downstream effects of this dysregulation, including on protein expression of target genes, as well as the affected signalling pathways are still poorly understood. We show there is a conserved region of approximately 500 bp of unmethylated DNA ±1 kb from the *CASR* TSS present in all three normal pancreatic islet cell types, which is lost in QGP‐1 cells. In addition, this chromatin region is in a closed confirmation in QGP‐1 cells. This indicates that the epigenetic alterations occurring in GEP‐NETs result in silencing of the *CASR* locus which leads to loss of CaSR protein expression, and therefore loss of normal CaSR signalling, which may play a role in reducing cellular proliferation.

Our finding that the CaSR is down‐regulated in gastrinomas, insulinomas, and NF‐PNETs suggests that common pathways are dysregulated in these tumours. At the genetic level, these tumour subtypes can differ, for example, *MEN1* mutations are rarely seen in insulinomas but occur in greater than 40% of NF‐PNETs.^8^ Furthermore, it has previously been reported that calcium plays an important role in hormone secretion, and a calcium stimulation test has been used to localise small insulinomas in clinical practice.^31^ In addition, the CaSR is highly expressed in pancreatic *α* and *β* cells and it has been shown that calcimimetic agents that act on the CaSR can stimulate glucagon and insulin secretion from pancreatic islets. Furthermore, mice with a gain‐of‐function CaSR mutation have impaired glucose tolerance.^32^, ^33^, ^34^ Based on this evidence, it may be expected that CaSR expression would be lost in non‐functioning tumours but maintained in functioning tumours. However, a direct link between CaSR signalling and insulin secretion in humans has not yet been demonstrated. If the CaSR was essential in insulin secretion it would be expected that its expression would be maintained in insulinomas. Our data showing reduced CaSR expression in insulinomas would suggest that insulin secretion is not dependent on CaSR signalling, and therefore, the CaSR may be playing additional roles in these tumours.

Our results demonstrate that the CaSR can significantly reduce the proliferation of NF‐PNET cells which is in keeping with previous publications reporting that the CaSR can regulate cell proliferation in normal pancreatic islet cells. For example, it has been shown that mice harbouring a gain‐of‐function CaSR mutation have reduced islet area, reduced *β*‐cell mass, and reduced *β*‐cell ki‐67 staining.^33^ In addition, it has been reported that in both mice and zebrafish, the CaSR is essential in regulating hyperaminoacidemia‐induced *α*‐cell proliferation, with *CASR* knockdown resulting in significantly reduced *α*‐cells.^35^ Interestingly, the regulation of *α*‐cell proliferation was reported to be via amino acid activation of the CaSR. To elicit its calcitropic effects, the CaSR is activated by the binding of calcium, leading to signalling via one of multiple G‐proteins, including G_*q*/11_, G_*i*/o_, or G_12/13_, to stimulate varying downstream effectors.^36^ However, the CaSR can also bind amino acids, which is thought to bias CaSR towards signalling via the ERK1/2 pathway, and consistent with this, it was reported that *α*‐cell proliferation was simulated via the CaSR‐Gq‐ERK1/2 pathway.^35^, ^37^ The CaSR is expressed in the gastrointestinal tract where it senses both nutrients, for example, calcium and magnesium, and aromatic amino acids, and therefore, the balance of downstream signalling pathways may be important for the normal functioning of these cells.^9^ The ERK1/2 signalling pathway has been reported to be perturbed in GEP‐NETs and is known to play a role in tumorigenesis, regulating cellular proliferation and apoptosis. Therefore, we propose that the CaSR may contribute to the tumourigenic process via its effects on the ERK1/2 signalling pathway.^2^

Our study does have some limitations. For example, we only examined the epigenetic landscape of the QGP1 cell line, which is a poorly differentiated and high‐grade NF‐PNET cell line, therefore it would be interesting to investigate other cell lines, for example, BON‐1 or NT3 in the future to see if the same mechanisms of CaSR silencing are present. Furthermore, it was not possible to confirm the exact origin of the gastrinomas we included in this study, that is, small intestine or pancreas which may affect the data variability. In addition, although we examine *CASR* expression in patient samples, our study does not include any in vivo work. A future study, for example using QGP1 cells in a mouse xenograft, would help further elucidate the role of CaSR in these tumours, and provide an opportunity to assess the effects of epigenetic modulating compounds, for example, the DNA methyltransferase inhibitor azacytidine to see if CaSR could be re‐expressed.

In conclusion, our study demonstrates that CaSR levels are reduced in multiple GEP‐NET subtypes through epigenetic mechanisms and that CaSR loss may contribute to increased cellular proliferation. Our findings further expand our knowledge of the role of the CaSR as a tumour suppressor and the epigenetic mechanisms underlying GEP‐NET biology.

## AUTHOR CONTRIBUTIONS

**Katherine A. English:** Conceptualization; data curation; writing – original draft; methodology; investigation; validation; writing – review and editing; formal analysis. **Michelle Goldsworthy:** Conceptualization; data curation; formal analysis; methodology; investigation; writing – original draft; writing – review and editing. **Brittannie Willis:** Data curation; writing – review and editing. **Kreepa G. Kooblall:** Data curation; writing – review and editing. **Shweta Birla:** Data curation; writing – review and editing. **Andreas Selberherr:** Writing – review and editing; resources. **Mark Stevenson:** Data curation; writing – review and editing. **Omair A. Shariq:** Data curation; writing – review and editing. **Ann L. Oberg:** Writing – review and editing; resources. **Tony Wang:** Writing – review and editing; formal analysis. **James Carmichael:** Writing – review and editing; resources. **Konstantinos Mavrommatis:** Writing – review and editing; resources. **Laure Escoubet:** Writing – review and editing; resources. **Rajesh V. Thakker:** Funding acquisition; resources; writing – review and editing. **Sarah A. Howles:** Funding acquisition; formal analysis; conceptualization; supervision; investigation; resources; writing – review and editing; writing – original draft. **Kate E. Lines:** Conceptualization; data curation; formal analysis; writing – original draft; writing – review and editing; funding acquisition; supervision.

## FUNDING INFORMATION

This work was supported by: the UK Medical Research Council (MRC) grants G9825289 and G1000467 (KEL, MS, RVT); Society for Endocrinology Summer Studentship grant (KEL, BW); Society for Endocrinology practical skills grant (SB, KEL); Oxford‐BMS fellowship (KEL); Cancer Research UK (CRUK) grant number C2195/A28699, through a CRUK Oxford Centre Clinical Research Training Fellowship (KE); a Wellcome Trust Senior Investigator Award (RVT); a Wellcome Trust Clinical Career Development Award (SAH), National Institute for Health Research (NIHR) Senior Investigator Award (RVT); and NIHR Oxford Biomedical Research Centre Programme (RVT). Sample collection was supported in part by the Mayo Clinic SPORE in Pancreatic Cancer (P50 CA102701) and the Lustgarten Foundation for Pancreatic Cancer Research.

## CONFLICT OF INTEREST STATEMENT

There are no conflicts of interest.

## ETHICS STATEMENT

All samples used in this study were obtained using protocols approved by local and national research ethics committees (approval number: 1053/2013).

## Supporting information


**Data S1:** Supporting Information

## Data Availability

The methylation array and ATAC‐seq data generated in this study are available in FigShare. Links to the data are as follows: methylation array https://figshare.com/s/a631a9ddcadea890b2c6; NF‐PNET cell lines ATAC‐Seq https://doi.org/10.6084/m9.figshare.26166457.v1 and Kidney cell lines ATAC‐Seq https://doi.org/10.6084/m9.figshare.26166454.v1. Other data that support the findings of this study are available from the corresponding author upon request.

## References

[1] Yao JC , Hassan M , Phan A , et al. One hundred years after "carcinoid": epidemiology of and prognostic factors for neuroendocrine tumors in 35,825 cases in the United States. J Clin Oncol. 2008;26:3063‐3072. doi:10.1200/jco.2007.15.4377 18565894

[2] Frost M , Lines KE , Thakker RV . Current and emerging therapies for PNETs in patients with or without MEN1. Nat Rev Endocrinol. 2018;14:216‐227. doi:10.1038/nrendo.2018.3 29449689 PMC6538535

[3] Mafficini A , Scarpa A . Genetics and epigenetics of Gastroenteropancreatic neuroendocrine neoplasms. Endocr Rev. 2019;40:506‐536. doi:10.1210/er.2018-00160 30657883 PMC6534496

[4] Scarpa A , Chang DK , Nones K , et al. Whole‐genome landscape of pancreatic neuroendocrine tumours. Nature. 2017;543:65‐71. doi:10.1038/nature21063 28199314

[5] Jiao Y , Shi C , Edil BH , et al. DAXX/ATRX, MEN1, and mTOR pathway genes are frequently altered in pancreatic neuroendocrine tumors. Science. 2011;331:1199‐1203. doi:10.1126/science.1200609 21252315 PMC3144496

[6] Li FA‐O , Deng Z , Zhang L , et al. ATRX loss induces telomere dysfunction and necessitates induction of alternative lengthening of telomeres during human cell immortalization. EMBO J. 2019;38(19):e96659. doi:10.15252/embj.201796659 PMC6769380 31454099

[7] Dyer MA , Qadeer ZA , Valle‐Garcia D , Bernstein E . ATRX and DAXX: mechanisms and mutations. Cold Spring Harb Perspect Med. 2017;7:a026567. doi:10.1101/cshperspect.a026567 28062559 PMC5334245

[8] Brandi ML , Agarwal SK , Perrier ND , Lines KE , Valk GD , Thakker RV . Multiple endocrine neoplasia type 1: latest insights. Endocr Rev. 2021;42:133‐170. doi:10.1210/endrev/bnaa031 33249439 PMC7958143

[9] Hannan FM , Kallay E , Chang W , Brandi ML , Thakker RV . The calcium‐sensing receptor in physiology and in calcitropic and noncalcitropic diseases. Nat Rev Endocrinol. 2018;15:33‐51. doi:10.1038/s41574-018-0115-0 30443043 PMC6535143

[10] Kim W , Takyar FM , Swan K , et al. Calcium‐sensing receptor promotes breast cancer by stimulating Intracrine actions of parathyroid hormone‐related protein. Cancer Res. 2016;76:5348‐5360. doi:10.1158/0008-5472.Can-15-2614 27450451 PMC5026591

[11] Bernichtein S , Pigat N , Barry Delongchamps N , et al. Vitamin D3 prevents calcium‐induced progression of early‐stage prostate tumors by counteracting TRPC6 and calcium sensing receptor upregulation. Cancer Res. 2017;77:355‐365. doi:10.1158/0008-5472.Can-16-0687 27879271

[12] Ahearn TU , Tchrakian N , Wilson KM , et al. Calcium‐sensing receptor tumor expression and lethal prostate cancer progression. J Clin Endocrinol Metab. 2016;101:2520‐2527. doi:10.1210/jc.2016-1082 27115058 PMC4891799

[13] Lines KE , Gluck AK , Thongjuea S , Bountra C , Thakker RV , Gorvin CM . The bromodomain inhibitor JQ1+ reduces calcium‐sensing receptor activity in pituitary cell lines. J Mol Endocrinol. 2021;67:83‐94. doi:10.1530/jme-21-0030 34223822 PMC8345903

[14] Casalà C , Gil‐Guiñón E , Ordóñez JL , et al. The calcium‐sensing receptor is silenced by genetic and epigenetic mechanisms in unfavorable neuroblastomas and its reactivation induces ERK1/2‐dependent apoptosis. Carcinogenesis. 2013;34:268‐276. doi:10.1093/carcin/bgs338 23108190

[15] Fetahu IS , Tennakoon S , Lines KE , et al. miR‐135b‐ and miR‐146b‐dependent silencing of calcium‐sensing receptor expression in colorectal tumors. Int J Cancer. 2016;138:137‐145. doi:10.1002/ijc.29681 26178670

[16] Lines KE , Nachtigall LB , Dichtel LE , et al. Multiple endocrine neoplasia type 1 (MEN1) Phenocopy due to a cell cycle division 73 (CDC73) variant. J Endocr Soc. 2020;4:bvaa142. doi:10.1210/jendso/bvaa142 33150274 PMC7594654

[17] Lines KE , Vas Nunes RP , Frost M , Yates CJ , Stevenson M , Thakker RV . A MEN1 pancreatic neuroendocrine tumour mouse model under temporal control. Endocr Connect. 2017;6:232‐242. doi:10.1530/ec-17-0040 28420716 PMC5632719

[18] Tian Y , Morris TJ , Webster AP , et al. ChAMP: updated methylation analysis pipeline for Illumina BeadChips. Bioinformatics. 2017;33:3982‐3984. doi:10.1093/bioinformatics/btx513 28961746 PMC5860089

[19] Sastre A , Valentino K , Hannan FM , et al. PTH infusion for seizures in autosomal dominant hypocalcemia type 1. N Engl J Med. 2021;385:189‐191. doi:10.1056/NEJMc2034981 34233101 PMC7614858

[20] Team, R. C . R: A Language and Environment for Statistical Computing. R Foundation for Statistical Computing.https://www.R-project.org/; 2022.

[21] Loyfer N , Magenheim J , Peretz A , et al. A DNA methylation atlas of normal human cell types. Nature. 2023;613:355‐364. doi:10.1038/s41586-022-05580-6 36599988 PMC9811898

[22] Exner S , Prasad V , Wiedenmann B , Grötzinger C . Octreotide does not inhibit proliferation in five neuroendocrine tumor cell Lines. Front Endocrinol. 2018;9:146. doi:10.3389/fendo.2018.00146 PMC5897986 29681888

[23] Lines KE , Stevenson M , Filippakopoulos P , et al. Epigenetic pathway inhibitors represent potential drugs for treating pancreatic and bronchial neuroendocrine tumors. Oncogenesis. 2017;6:e332. doi:10.1038/oncsis.2017.30 28504695 PMC5523063

[24] Pfaffl MW . A new mathematical model for relative quantification in real‐time RT‐PCR. Nucleic Acids Res. 2001;29:e45. 11328886 10.1093/nar/29.9.e45 PMC55695

[25] Luley KB , Biedermann SB , Künstner A , et al. A comprehensive molecular characterization of the pancreatic neuroendocrine tumor cell lines BON‐1 and QGP‐1. Cancers. 2020;12(3):691. doi:10.3390/cancers12030691 PMC7140066 32183367

[26] Hannan FM , Gorvin CM , Babinsky VN , et al. Calcilytic NPSP795 increases plasma calcium and PTH in an autosomal dominant hypocalcemia type 1 mouse model. JBMR Plus. 2020;4:e10402. doi:10.1002/jbm4.10402 33103030 PMC7574706

[27] Gao Y , Robertson MJ , Rahman SN , et al. Asymmetric activation of the calcium‐sensing receptor homodimer. Nature. 2021;595:455‐459. doi:10.1038/s41586-021-03691-0 34194040 PMC8826748

[28] Gorvin CM , Frost M , Malinauskas T , et al. Calcium‐sensing receptor residues with loss‐ and gain‐of‐function mutations are located in regions of conformational change and cause signalling bias. Hum Mol Genet. 2018;27:3720‐3733. doi:10.1093/hmg/ddy263 30052933 PMC6196656

[29] Forcet C , Ye X , Granger L , et al. The dependence receptor DCC (deleted in colorectal cancer) defines an alternative mechanism for caspase activation. Proc Natl Acad Sci USA. 2001;98:3416‐3421. doi:10.1073/pnas.051378298 11248093 PMC30668

[30] Sharma R , Lythgoe MP , Slaich B , Patel N . Exploring the epigenome in gastroenteropancreatic neuroendocrine neoplasias. Cancers. 2021;13(16):4181. doi:10.3390/cancers13164181 PMC8394968 34439335

[31] Guettier JM , Kam A , Chang R , et al. Localization of insulinomas to regions of the pancreas by intraarterial calcium stimulation: the NIH experience. J Clin Endocrinol Metab. 2009;94:1074‐1080. doi:10.1210/jc.2008-1986 19190102 PMC2682461

[32] Gray E , Muller D , Squires PE , et al. Activation of the extracellular calcium‐sensing receptor initiates insulin secretion from human islets of Langerhans: involvement of protein kinases. J Endocrinol. 2006;190:703‐710. doi:10.1677/joe.1.06891 17003271

[33] Babinsky VN , Hannan FM , Ramracheya RD , et al. Mutant mice with calcium‐sensing receptor activation have hyperglycemia that is rectified by calcilytic therapy. Endocrinology. 2017;158:2486‐2502. doi:10.1210/en.2017-00111 28575322 PMC5551547

[34] Straub SG , Kornreich B , Oswald RE , Nemeth EF , Sharp GW . The calcimimetic R‐467 potentiates insulin secretion in pancreatic beta cells by activation of a nonspecific cation channel. J Biol Chem. 2000;275:18777‐18784. doi:10.1074/jbc.M000090200 10751384

[35] Gong Y , Yang B , Zhang D , et al. Hyperaminoacidemia induces pancreatic α cell proliferation via synergism between the mTORC1 and CaSR‐Gq signaling pathways. Nat Commun. 2023;14:235. doi:10.1038/s41467-022-35705-4 36646689 PMC9842633

[36] Abid HA , Inoue A , Gorvin CM . Heterogeneity of G protein activation by the calcium‐sensing receptor. J Mol Endocrinol. 2021;67:41‐53. doi:10.1530/jme-21-0058 34077389 PMC8240730

[37] Thomsen AR , Hvidtfeldt M , Bräuner‐Osborne H . Biased agonism of the calcium‐sensing receptor. Cell Calcium. 2012;51:107‐116. doi:10.1016/j.ceca.2011.11.009 22192592

