# Supplementary material for: Calcium sensing receptor expression is downregulated in gastroenteropancreatic neuroendocrine tumours via epigenetic mechanisms
Source: Int J Cancer. 2024 Nov 23;156(5):980–92. doi: 10.1002/ijc.35264 (PMC11701399; doi:10.1002/ijc.35264)
Supplement: Supplementary file 1 — Data S1: Supporting Information [file IJC-156-980-s001.pdf]

Calcium Sensing receptor expression is downregulated in gastroenteropancreatic neuroendocrine tumours via epigenetic mechanisms.

Katherine A. English, Michelle Goldsworthy, Brittannie Willis, Kreepa G. Kooblall, Shweta Birla, Andreas Selberherr, Mark Stevenson, Omair A. Shariq, Ann L. Oberg, Tony Wang, James Carmichael, Konstantinos Mavrommatis, Laure Escoubet, Rajesh V. Thakker, Sarah A. Howles & Kate E. Lines

Contents:

Supplementary Table 1. Quality statistics for EPIC array DNA methylation profiling.

Supplementary Table 2. Quality statistics for QGP1 single cell assay for transposase-accessible chromatin using sequencing (ATAC-Seq) data.

Supplementary Figure 1. H&E and synaptophysin expression in cohort of GEP-NET FFPE samples.

Supplementary Figure 2. Analysis of CaSR transfection on HEK293T cell viability

Supplementary Table 1. Quality statistics for EPIC array DNA methylation profiling. CpG probes loaded and filtered in R using R ChAMP pipeline. GRCh38/hg38 reference genome was used.

Cell line	Total number of probes	Fraction of failed positions per sample	Total number of probes for analysis	Probes removed					
				Detection p-vale >0.01	Beadcount <3 in at least 5% of samples.	NoCG Start	Probes identified in Zhou et al., 2017[4]	Multi-hit start Nordlund et al., 2013[6]	Located on XY chromosome
QGP1	865,918	0.019	731,586	17,617	1,975	2,822	95,872	11	16,035

Supplementary Table 2. Quality statistics for QGP1 single cell assay for transposase-accessible chromatin using sequencing (ATAC-Seq) data. GRCh38/hg38 reference genome was used. Column headings refer to Cell Ranger Software (10X Genomics) metrics, for example frac_fragments_overlapping_peaks refers to the fraction of overlapping peaks and frac_waste_mitochondrial refers to the fraction of mitochondrial reads filtered out.

cells_detected	frac_fragments_overlapping_peaks	frac_mapped_confidently	frac_waste_mitochondrial	frac_waste_total	median_fragments_per_cell	total_usable_fragments	tss_enrichment_score
5129	59.8%	89.2%	0.2%	69.4%	15546	1.05E+08	6.49859

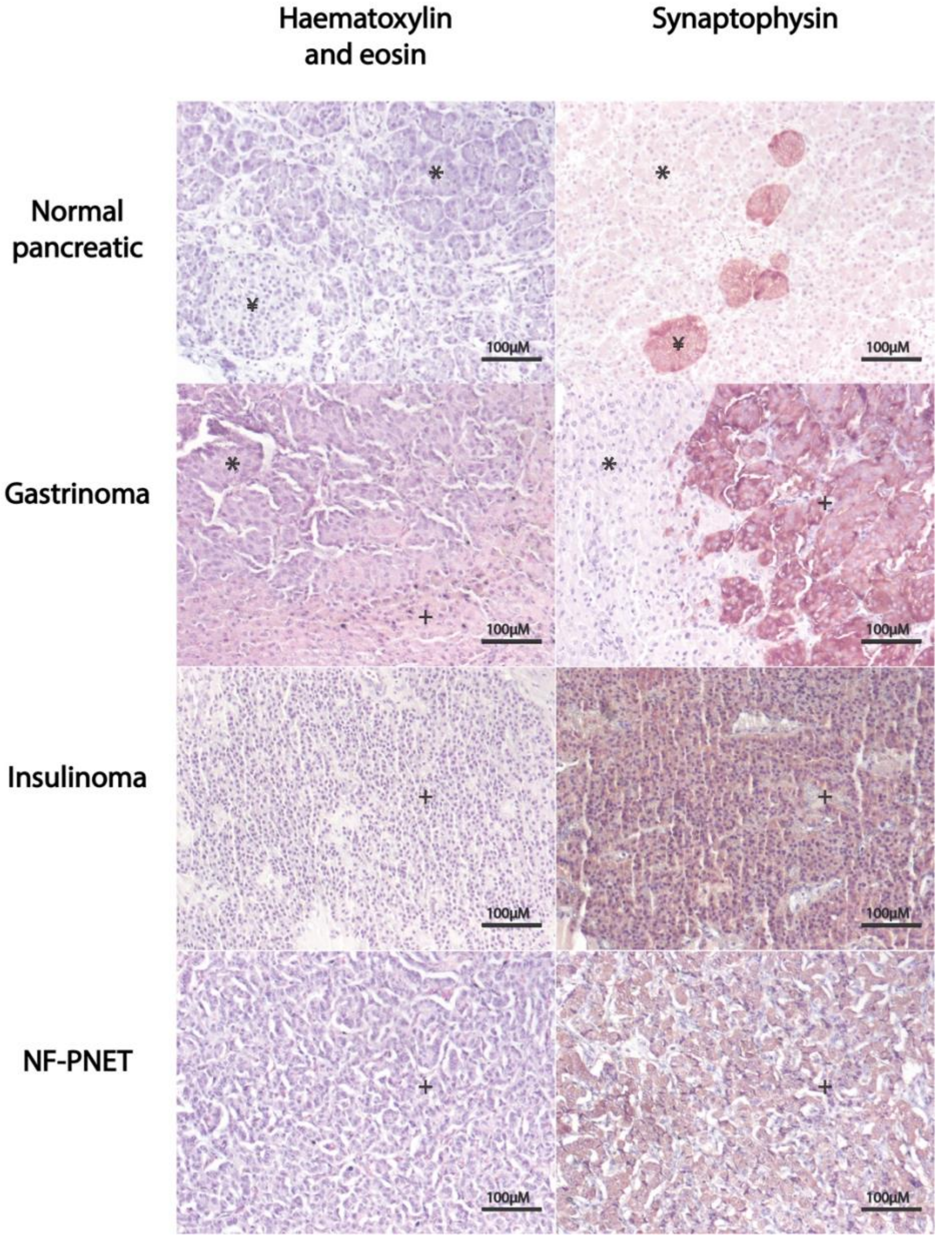

Supplementary Figure 1. H&E and synaptophysin expression in cohort of GEP-NET FFPE samples. Representative images are shown for each GEP-NET subtype examined, as well as normal pancreatic islets. Positive staining is shown in brow. Normal islets (¥), exocrine pancreatic tissue (*) and islet cell tumours (+). Scale bar represents 100μm.

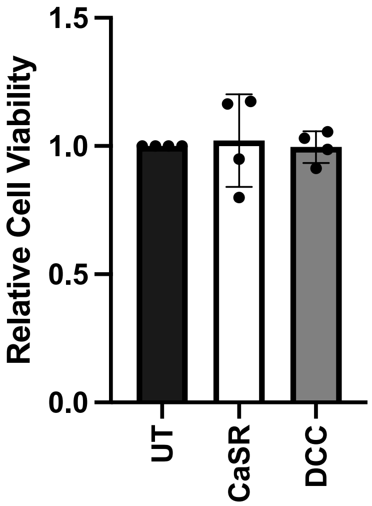

Supplementary Figure 2. Analysis of CaSR transfection on HEK293T cell viability. The effect of CaSR expression on cell viability was assessed using Cell Titer Blue assay. Viability of HEK293T cells transfected with CaSR or DCC was assessed after 96 hours, untransfected cells (UT) were used as a control. Data is represented relative to UT cells.
